# Water, Alcohols, Amines, and Amides as Formal Hydrogen‐Atom‐Transfer Reagents Through Activation With Redox‐Active Lewis Acids

**DOI:** 10.1002/anie.8159070

**Published:** 2026-08-05

**Authors:** Petra Vojáčková, Armido Studer

**Affiliations:** ^1^ Organisch‐Chemisches Institut Universität Münster Münster Germany

**Keywords:** bond weakening, hydrogen‐atom donors, hydrogen‐atom transfer, proton‐coupled electron transfer, radical chemistry

## Abstract

The O─H and N─H bonds of protic molecules such as water, alcohols, amines, or amides typically resist hydrogen‐atom abstraction because of the high energy of the resulting heteroatom‐centered radical intermediates. However, association of the protic molecules with redox‐active Lewis acids provides stabilization of the generated reactive radical intermediates and enables the ligated protic molecules to serve as hydrogen‐atom donors or proton‐coupled‐electron‐transfer reagents. This review presents an overview of applications of this bond‐weakening principle in organic synthesis and attempts to illustrate the breadth of systems that enable such reactivity.

## Graphical abstract

Protic molecules containing strong O─H or N─H bonds typically resist hydrogen‐atom abstraction because of the high reactivity of the resulting heteroatom‐centered radicals. However, association of the protic molecules with redox‐active Lewis acids can provide powerful hydrogen‐atom donors or proton‐coupled‐electron‐transfer reagents. The review highlights the variety of systems enabling this reactivity and summarizes their synthetic applications.

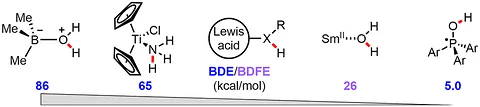

## 1 Introduction

**FIGURE 1:**
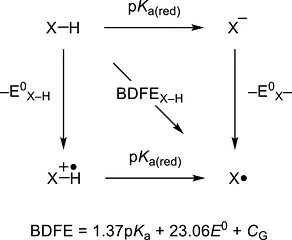
Square scheme of PCET thermochemistry.

Proton‐coupled electron transfer (PCET) reactions are fundamental steps in radical chemistry that either generate free radical intermediates from closed‐shell substrates or facilitate termination of radical intermediates by creating a new bond to hydrogen [1]. Hydrogen‐atom transfer (HAT) represents a subclass of PCET reactions that describes a concerted movement of an electron and a proton occupying the same donor and acceptor orbitals [2]. By contrast, a concerted movement of a proton and an electron originating in or being transferred to two different orbitals is classified as a multisite proton‐coupled electron transfer (MS‐PCET). The separation of the origin of the proton and the electron in MS‐PCET reagents enables extensive tuning of both the p*K*_a_ and reduction potential of the reagent, thus enabling generation of products with even very weak newly formed bonds to hydrogen such as those adjacent to an unpaired electron [1, 3]. The formal bond‐dissociation free energy (BDFE) of a PCET reagent can be calculated using the Bordwell thermodynamic equation that includes the p*K*_a_ and the reduction potential of the reagent (Figure 1), where *C*_G_ represents a solvent dependent constant that accounts for the energy required to convert a proton and an electron to a hydrogen atom [4].

Water generally represents a poor hydrogen‐atom donor because of the strong O─H bonds with bond‐dissociation energy (BDE) of 117.6 kcal mol^−1^ (equals to BDFE of 114.7 kcal mol^−1^) [5, 6]. However, association of water with certain redox‐active Lewis acids, mostly low‐valent metal complexes, enables water to act as a potent PCET reagent in radical reactions [4, 6]. For instance, coordination of water to a tetranuclear manganese cluster in photosystem II has been proposed to enable a formal hydrogen‐atom transfer from water in nature, releasing dioxygen as a by‐product [7, 8]. One of the largest reported O─H‐bond weakening of water was achieved with samarium(II) dichloride, which enabled reduction of substrates through formation of C─H bonds with BDFE of 19.5–25.1 kcal mol^−1^ [9].

**FIGURE 2:**
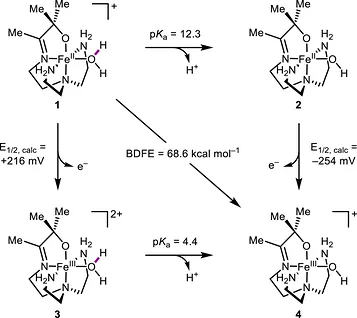
Thermochemical square scheme for Fe(II)‐derived system developed by Kovacs and coworkers (redox potential vs. NHE).

The extent of bond weakening is directly related to the difference in binding energies of water and the corresponding hydroxyl radical to the redox‐active Lewis acid [6]. The importance of both the p*K*_a_ and the reducing ability of the activator for inducing a bond‐weakening effect was demonstrated by Kovacs and co‐workers (Figure 2) [10]. Coordination of water to iron(II) complex to form **1** was determined to increase the acidity of water by three orders of magnitude. Oxidation of **1** to the corresponding iron(III) species **3** was observed to further increase the acidity of water of approximately eight orders of magnitude. The corresponding oxidation potentials were calculated using the Bordwell equation together with known p*K*_a_ and BDFE values. The analysis revealed that electron removal from protonated complex **1** was nearly 11 kcal mol^−1^ less favorable than from the deprotonated complex **2**, providing iron(III) complex **4**. Overall, these results demonstrate that the ability of a Lewis acid to induce bond weakening in coordinated ligands depends on both its Lewis acidity and its reducing power.

**FIGURE 3:**
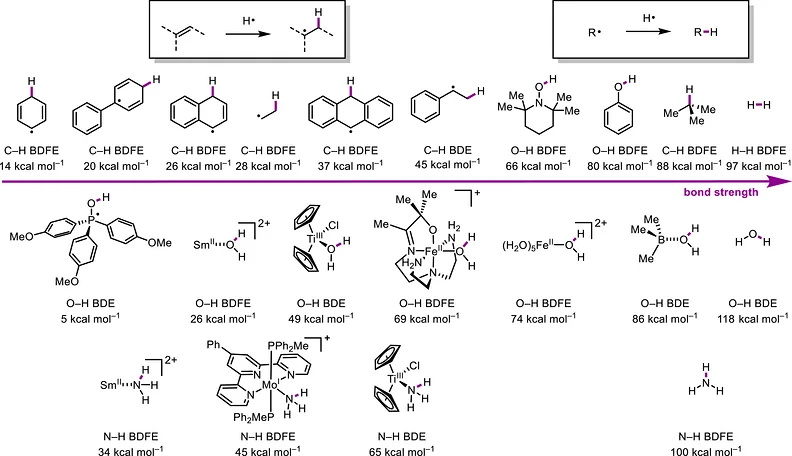
O─H/N─H‐bond weakening effects induced by redox‐active Lewis acids.

The coordination‐induced bond weakening effect is not limited to water as other protic small molecules such as alcohols, phenols, amines, or amides have been demonstrated to serve as a source of hydrogen atoms upon coordination to redox‐active Lewis acids [11]. The selection of the specific combination of the protic ligand and the redox‐active Lewis acid allows for modulation of the O─H/N─H‐bond strength to match the desired application (Figure 3).

This review aims to provide an overview of chemical systems in which small protic molecules containing strong O─H or N─H bonds act as formal hydrogen‐atom donors (HAT or MS‐PCET reagents) upon association with redox‐active Lewis acids. Multiple reviews and perspectives already covered the topic of coordination‐induced bond weakening [3, 4, 6, 11, 12, 13, 14, 15]. The review by Boekell and Flowers provides a detailed introduction to the topic of coordination‐induced bond weakening and includes reports until 2022 [11]. Our review is not intended to extensively cover all examples of this phenomenon reported in the literature but instead, the aim is to highlight the diversity of systems that enable weakening of strong protic bonds and the applications of this concept in organic synthesis using selected examples. Although the bond‐weakening effect was originally described on systems in which protic molecules associate with redox‐active Lewis acids through weak coordination, we decided to include also systems that form covalent bonds between protic molecules and redox‐active Lewis‐acids to demonstrate the mechanistic diversity of strategies for activation of strong protic bonds for formal hydrogen‐atom‐transfer reactions. Our discussion is focused only on homogenous systems that induce a formal transfer of hydrogen atoms from either O─H or N─H bonds. Heterogeneous systems were not included in this review, because a number of different experimental techniques is required for their study when compared to homogeneous systems [16, 17, 18, 19, 20, 21, 22, 23]. The review covers only examples of PCET reactivity of ground‐state systems generated by association of simple protic molecules. Excitation of metal complexes containing protic ligands with light can also trigger PCET reactivity, but precoordination of the protic molecule is usually required in these systems due to short lifetimes of the excited redox‐active Lewis acid species [24, 25, 26, 27, 28]. In addition, excited‐state PCET has been reported mainly in the context of more complex ligands containing protic functionalities rather than coordinated simple protic molecules. The review is divided into chapters according to the central atom of the Lewis acid that enables weakening of O─H or N─H bonds.

## 2 Weakening of Protic Bonds by Systems Containing Redox‐Active Main‐Group Elements

### 2.1 Boron

**FIGURE 4:**
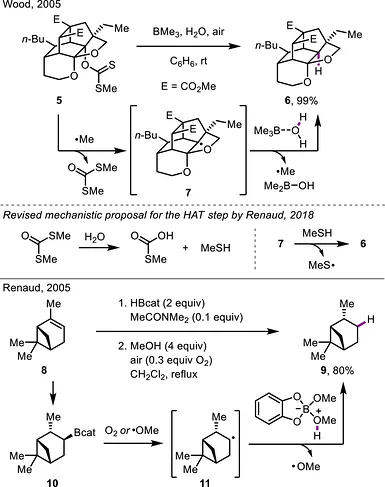
Reduction of xanthate esters in trialkylborane‐mediated Barton–McCombie reactions with water as the source of a hydrogen atom. Reduction of alkylcatecholborane intermediates with borate‐activated methanol according to the original mechanistic proposal.

In 1990, Barton, Jang, and Jaszberenyi observed that a tin hydride reagent was not necessary for reductions of xanthate esters to the corresponding alkane products in trialkylborane‐mediated variants of the Barton‐McCombie reaction [29]. However, the identity of the hydrogen‐atom source was not suggested at that time. In 2005, Wood and coworkers discovered in the course of their synthetic studies toward phomoidride natural products that water could serve as the hydrogen‐atom source in this type of reduction reactions (Figure 4) [30]. The structurally complex xanthate **5** was efficiently hydrodefunctionalized to **6** using trimethylborane and water under air atmosphere. The use of D_2_O instead of H_2_O enabled the preparation of the corresponding product with 94% deuterium incorporation. Direct HAT from water was suggested as an unlikely mechanism because of the high strength of the O─H bond in water. Instead, the authors proposed that activation of water by coordination to the Lewis acidic borane reagent enabled water to act as the hydrogen‐atom donor. Indeed, computational studies suggested that coordination of water to trimethylborane decreased the BDE of the O─H bond of water by 30 kcal mol^−1^. A concerted fragmentation during HAT providing a methyl radical along with Me_2_BOH was identified as an important contribution to the bond‐weakening effect, responsible for an additional driving force of 13 kcal mol^−1^ [31]. Overall, the reaction was proposed to be initiated by oxidation of trialkylborane with air yielding an alkyl radical. Addition of the alkyl radical to the xanthate group of a substrate was suggested to trigger a β‐fragmentation to form substrate‐derived tertiary alkyl radical **7** that upon reduction with the borane‐water complex afforded the hydrodefunctionalized product and another equivalent of an alkyl radical capable of propagating the chain reaction.

The original mechanistic proposal made by Wood in 2005 was later re‐evaluated by Renaud and coworkers, who suggested that hydrogen‐atom transfer from water–borane complexes represented only a minor pathway in the reduction of xanthate‐derived substrates [32]. A thiol catalyst generated in situ by fragmentation of the xanthate group was proposed to be responsible for mediating reductions of substrate‐derived radical intermediates to the corresponding alkane products. This mechanistic proposal was consistent with the observations that the level of deuteration using the borane–water conditions depended on the substrate used. When alkyl iodides were used as the source of alkyl radicals, only a small level of deuteration was observed, while almost complete deuteration was measured with the corresponding xanthate substrates. The addition of 1 mol % of a thiol catalyst to the reaction of alkyl iodide substrates resulted in higher deuterium incorporation (81% in contrast to 7% in the absence of the thiol), which supported the role of the thiol catalyst in reductions of xanthate substrates.

Almost at the same time as Wood, Renaud, and coworkers independently reported that alkylcatecholboranes (**10**) generated by hydroboration of alkene substrates (**8**) were reduced to the corresponding alkane products (**9**) in the presence of alcohols and air or peroxides (Figure 4) [33]. Based on control and deuteration experiments, the authors proposed that oxygen‐induced fragmentation of alkylcatecholboranes afforded alkyl radicals (**11**) that were reduced through a HAT from MeOBcat–methanol complexes [34]. However, subsequent mechanistic studies by the same group revealed that catechol was generated in the reaction mixture by methanolysis of catecholboranes, which was identified as a more likely hydrogen‐atom donor for reductions of alkyl radicals than the originally proposed MeOBcat–methanol complex [35].

Jin and Newcomb studied the rate of HAT from a BEt_3_–water complex through radical‐clock experiments [36, 37]. They found that the BEt_3_–water complex reacted with secondary alkyl radicals in benzene at 20 °C with a rate constant around two orders of magnitude smaller than tin hydride reagents. Variable temperature studies with the Et_3_B–methanol complex in toluene indicated that the HAT reaction had an unusually high entropic demand, which resulted in substantially more efficient HAT trapping reactions in competition with radical ring‐opening and cyclization reactions at reduced temperatures [37]. Nuclear‐magnetic‐resonance (NMR) studies by Renaud and coworkers later revealed that only 1%–2% of triethylborane existed as the complex with methanol under most of the reported reaction conditions, which suggested that HAT to secondary alkyl radicals from the Et_3_B–methanol complex was an even more efficient process with a rate constant in the range of 10^6^ M^−1^ s^−1^ [38].

**FIGURE 5:**
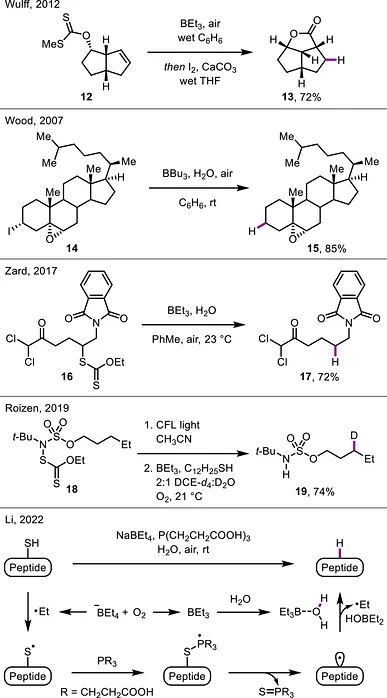
Applications of borane–water/alcohol combinations in synthesis. CFL = compact fluorescent light.

The use of trialkylborane–water complexes as hydrogen‐atom source for reductions of alkyl radicals was extended to radical cyclization reactions (see **12** to **13**, Figure 5) [39], reductions of alkyl iodides (**14**) and *S*‐alkyl‐thionocarbonates to give the corresponding hydro‐defunctionalized products (**15**) [32, 40, 41, 42], and found other synthetic applications [43]. Selective reductions of xanthate esters in the presence of a sensitive α,α‐dichloroketone group was achieved using the borane–water protocol by Anthore–Dalion and Zard (see **16** to **17**, Figure 5) [44]. Ayer and Roizen utilized the reductive conditions for a two‐step directed deuteration of sulfamate esters as exemplified in conversion of **18** to **19** (Figure 5) [45]. The mild conditions for alkyl radical reductions with borane–water complexes enabled the development of a chemoselective method for radical desulfurization of proteins using a tetraethylborate salt, a phosphine, water, and air at ambient temperature (Figure 5) [46]. The process was proposed to include a hydrogen‐atom transfer from a Et_3_B–water complex to alkyl radicals generated by a β‐fragmentation of phosphoranyl radical intermediates derived from thiol substrates.

### 2.2 Germanium

**FIGURE 6:**
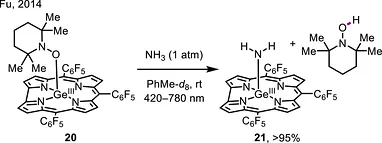
Weakening of N─H‐bonds of ammonia with a transient Ge(II)–corrole complex.

Fu and coworkers reported germanium(III)–corrole complex **20** carrying a (2,2,6,6‐tetramethylpiperidin‐1‐yl)oxyl (TEMPO) ligand, which upon light irradiation underwent a Ge─O bond homolysis (Figure 6) [47, 48]. The resulting tetracoordinated germanium(II) radical induced bond weakening of N─H and O─H bonds of protic substrates. In the presence of ammonia, formation of germanium(III)–amide complex **21** and TEMPO─H was observed. Computational and EPR studies of the germanium‐derived complex suggested that the electron was mostly located on the corrole ligand, consistent with the observed reactivity occurring through a PCET mechanism. The mechanism of ammonia activation through coordination to the germanium(II) complex derived from **20** was supported by the observation that methane did not promote a formal hydrogen‐atom transfer despite having relatively weak C─H bonds (BDFE of 105 kcal mol^−1^) in contrast to effective reagents containing Lewis basic sites such as water (BDFE of 119 kcal mol^−1^), methanol (105 kcal mol^−1^), or ammonia (108 kcal mol^−1^) [48].

### 2.3 Bismuth

**FIGURE 7:**
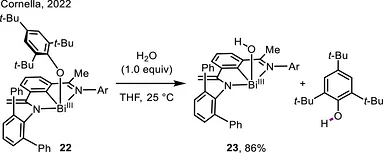
Weakening of O─H bonds of water with a transient Bi(II) complex.

In 2022, Cornella and coworkers disclosed a radical equilibrium complex based on bismuth(III) (**22**) containing an extremely weak Bi─O bond (Figure 7) [49]. Homolysis of the Bi─O bond at room temperature revealed a bismuth(II) center with a radical character that demonstrated coordination‐induced‐bond‐weakening properties toward amines, alcohols, and water. Hydrogen‐atom transfer to the in situ‐generated phenoxyl radical yielded the corresponding amido‐ and alkoxy‐bismuth(III) complexes (**23**). Computational analysis of the developed system revealed a large bond‐weakening effect induced by the bismuth(II) radical center on water (BDFE of 52.1 kcal mol^−1^) and ammonia (BDFE of 47.0 kcal mol^−1^).

### 2.4 Phosphorus

**FIGURE 8:**
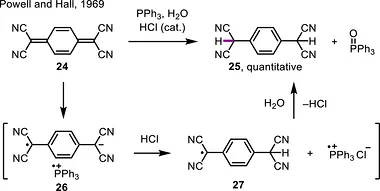
Formal hydrogenation with water and phosphines.

To our knowledge, the first report demonstrating the ability of phosphines to activate water for formal hydrogen‐atom transfer to substrates was disclosed in 1969 by Powell and Hall (Figure 8) [50]. Triphenylphosphine was found to react with 7,7,8,8‐tetracyanoquinodimethane (**24**) in the presence of water and catalytic quantities of HCl, providing **25** and triphenylphosphine oxide in a quantitative yield. It was proposed that triphenylphosphine and **24** formed an electron donor–acceptor (EDA) complex or radical ion pair **26**. Subsequent reaction with HCl was suggested to provide radical **27** and phosphine radical cation–chloride ion pair. Activation of water with the phosphine radical cation was hypothesized to enable the reduction of radical **27**, while releasing triphenylphosphine oxide. The authors observed that the reaction rate varied depending on the nature of the aryl substituents of phosphines. Specifically, electron‐rich arene groups were observed to increase the reaction rate. Although the authors did not propose a specific mechanism of water activation by the phosphine radical cation in their original study, their experimental observations are consistent with the intermediacy of water‐derived phosphoranyl radicals. Water‐derived phosphoranyl radicals were first postulated by Pandey and coworkers in 1991, who showed that water can react with photocatalytically generated phosphine radical cations yielding the corresponding phosphine oxide products [51].

**FIGURE 9:**
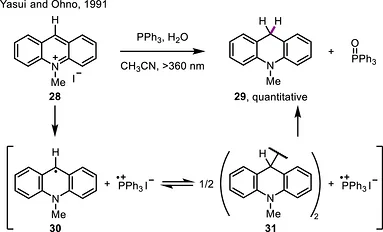
Formal hydrogenation with phosphines and water.

In the same year, Yasui and Ohno reported a formal hydrogenation of 10‐methylacridinium **28** under visible‐light irradiation in the presence of triphenylphosphine and water, resulting in the formation of 10‐methylacridan (**29**) and triphenylphosphine oxide in a quantitative yield (Figure 9) [52]. The authors proposed that the excited state of **28** underwent a single‐electron transfer with the phosphine, generating 10‐methylacridinyl radical (**30**) and the corresponding phosphine radical cation. Radical **30** was proposed to undergo dimerization to form 10,10′‐dimethyl‐9,9′‐biacridine (**31**), which could be detected in the reaction mixture during the process. Reduction of dimer **31** to product **29** was hypothesized to proceed through a single‐electron transfer from the phosphine to the excited state of **31**, yielding **29** upon protonation together with **30** and the corresponding phosphine radical cation. Although the authors did not suggest a specific role of water in the mechanism, the intermediacy of a water‐derived phosphoranyl radical acting as a PCET reagent in the reduction process cannot be ruled out.

**FIGURE 10:**
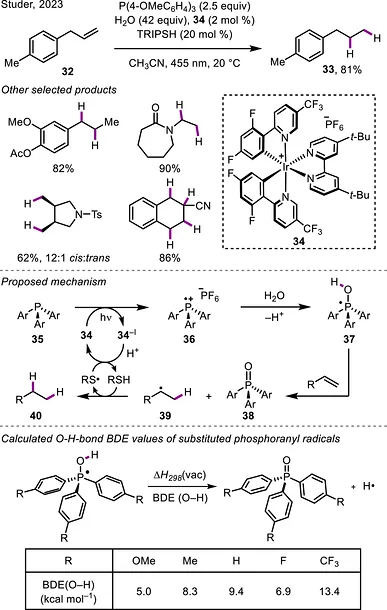
Radical hydrogenation of alkenes and naphthalenes with in‐situ‐generated phosphoranyl radicals. Proposed mechanism and calculated O─H‐bond BDE values of substituted phosphoranyl radicals (density functional theory (DFT): PWPB95‐D3//PBE0‐D3/def2‐TZVP + COSMO‐RS (CH_3_CN)). TRIPSH = 2,4,6‐triisopropylbenzenethiol.

In 2023, Studer and coworkers developed a mild protocol for formal hydrogenation of closed‐shell π‐systems using phosphines and water as the hydrogen‐atom source (see **32** to **33**, Figure 10) [53]. The reported system consisted of iridium(III) photoredox catalyst **34** that was proposed to undergo a visible‐light‐induced single‐electron transfer with phosphine **35**. Trapping of the resulting phosphine radical cation **36** with water present in the reaction mixture was suggested to form the reactive phosphoranyl radical **37** upon proton transfer. A formal transfer of a hydrogen atom from **37** to alkene substrates was proposed to yield radical **39** that was then reduced by an arylthiol catalyst to give hydrogenated product **40**. The resulting thiyl radical was suggested to be reduced by iridium(II)‐catalyst **34^−I^** to the thiolate anion and then protonated, thus closing the redox catalysis cycle. Formation of a strong P═O bond in the resulting phosphine oxide by‐product **38** was suggested as the main thermodynamic driving force for the efficient transfer of hydrogen atoms from the proposed phosphoranyl radicals to generate reactive C‐radicals. The radical character of the developed transformation was further supported by radical‐clock studies and radical cascade reactions of diene substrates (Figure 10).

Phosphines are well‐established ligands in transition‐metal catalysis. Their steric and electronic effects enable fine‐tuning of the reactivity of the corresponding P‐ligated metal catalysts [54]. Remarkably, the HAT reactivity of phosphoranyl radicals was also demonstrated to be modulated through electronic tuning of the aryl substituents on phosphorus (Figure 10). Computed O─H‐bond BDE values of selected triaryl phosphoranyl radicals were shown to span a range of 8 kcal mol^−1^. Tris(4‐methoxyphenyl)phosphine exhibited the lowest O─H‐bond BDE of only 5.0 kcal mol^−1^, whereas the least electron‐rich tris(4‐(trifluoromethyl)phenyl)phosphine showed a considerably higher O─H‐bond BDE of 13.4 kcal mol^−1^. However, basic phosphines such as Buchwald‐type ligands are not suitable reagents for hydrogenation reactions, as the corresponding phosphoranyl radicals were observed to be readily deprotonated under the basic reaction conditions. Deprotonation of phosphoranyl radicals was reported to lead to formation of phosphine oxide radical anions, which were shown to act as very strong single‐electron‐transfer (SET) reductants [55].

Lv, Lu, and coworkers studied the radical hydrogenation of alkene and naphthalene substrates with water‐derived phosphoranyl radicals computationally [56]. The authors suggested that a concerted MS‐PCET pathway rather than an HAT mechanism was operative in the hydrogenation process. Spin density of the phosphoranyl radical was predominantly localized on the phosphorus center, while the hydrogen associated with the oxygen atom of the P─O group carried a significant partial positive charge. Intrinsic bond orbital analysis of the hydrogenation step revealed transfer of electron density from the aryl group of the phosphoranyl radical to the π*‐orbital of the alkene substrate in concert with the proton transfer from the oxygen atom to the substrate. The proposed concerted MS‐PCET mechanism was shown to align well with the regioselectivity observed in hydrogenations of substituted naphthalene substrates in the original study by Studer and coworkers [53].

The combination of a phosphine and water in a redox‐mediated environment was employed also in other hydrogenation reactions of closed‐shell π‐systems [57, 58]. Control experiments demonstrated that the hydrogen atoms in products resulted from water. However, alternative mechanisms were suggested for the observed reactivity that did not include water‐derived phosphoranyl radicals.

**FIGURE 11:**
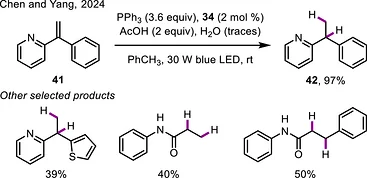
Hydrogenation of electron poor terminal alkenes in the absence of a thiol cocatalyst.

Chen, Yang, and coworkers reported an alternative protocol for hydrogenation of alkenes with phosphoranyl radicals, which omitted the use of a thiol HAT cocatalyst and instead incorporated an additional proton source in the form of acetic acid (Figure 11) [59]. It was found that trace amounts of water present in the solvents were sufficient to promote the reaction. By contrast, large amounts of externally added water were found to inhibit the process. Electron‐deficient alkene substrates, mostly vinyl pyridines (**41**) or acrylamides, were successfully converted to the corresponding hydrogenated products (**42**). Mechanistically, substrate radical intermediates generated through an HAT to the corresponding alkenes with water‐derived phosphoranyl radicals were proposed to be reduced by the reduced form of the iridium photocatalyst **34**. The resulting stabilized carbanion intermediates were suggested to provide the desired products upon protonation with acetic acid.

**FIGURE 12:**
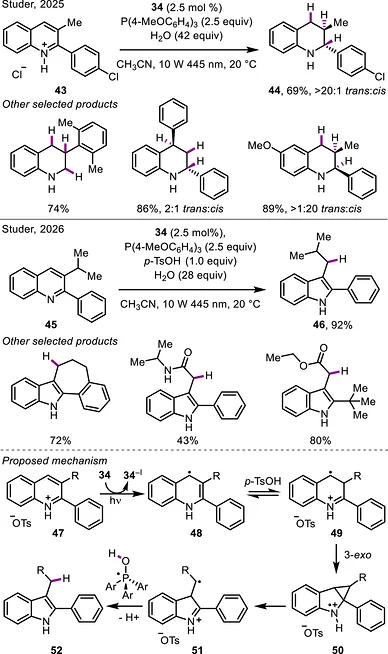
Partial hydrogenation of quinolines to 1,2,3,4‐tetrahydroquinolines and reductive skeletal editing of quinolines to indoles.

Water‐derived phosphoranyl radicals can also be applied to reductions of heteroarenes. In 2025, Studer and coworkers reported an extension of their protocol addressing semihydrogenations of quinolinium hydrochloride salts (**43**) to give 1,2,3,4‐tetrahydroquinolines (**44**) (Figure 12) [60]. In this process, the water‐derived phosphoranyl radical was proposed to transfer a hydrogen atom to a substrate C‐radical generated by a single‐electron reduction of the quinolinium salt. The second equivalent of a hydrogen atom was proposed to be transferred to the α‐amino alkyl radical, resulting from single‐electron reduction of the iminium intermediate. Initially generated *cis*‐hydrogenated products were observed to undergo photocatalytic epimerization to yield the corresponding *trans*‐isomers as major products (**44**), which are usually inaccessible through traditional transition‐metal‐catalyzed hydrogenation reactions. Interestingly, electron‐rich tetrahydroquinoline products did not undergo the epimerization process and were isolated exclusively as the kinetic *cis*‐isomers.

Very recently, the Studer group disclosed a selective reductive rearrangement of 2,3‐disubstituted quinolines (**45**) into 2,3‐disubstitued indoles (**46**) using phosphines and water under photocatalytic conditions (Figure 12) [61]. The proposed mechanism included quinoline protonation with tosic acid, yielding tosylate salt **47**, which was reduced through a single‐electron transfer from the photoexcited iridium catalyst **34**, as supported by Stern–Volmer quenching studies. Protonation of the resulting enamine radical intermediate **48** provided the distonic iminium radical cation **49** that was suggested to undergo a 3‐*exo* cyclization to generate the strained aminium radical cation **50**. Ring opening of **50** was proposed to lead to radical cation **51** that upon a hydrogen‐atom transfer and a deprotonation provided the rearranged indole product **52**.

**FIGURE 13:**
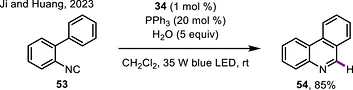
HAT‐initiated cyclization of 2‐isocyanobiphenyls to phenanthridines using a catalytic amount of triphenylphosphine.

The isonitrile functionality is another intriguing moiety that can be reduced with phosphoranyl radicals. In contrast to formal hydrogen‐atom additions to C═C and C═X double bonds, HAT to the isonitrile group generates an imidoyl radical with the new C─H bond and the open‐shell center at the same carbon. Ji, Huang, and coworkers applied this reactivity in cyclizations of 2‐isocyanobiphenyls (**53**) to phenanthridines (**54**) promoted by catalytic amounts of phosphines or phosphine oxides, an iridium complex, and water (Figure 13) [62]. The authors observed that phosphine oxides could serve as catalysts in place of phosphines and proposed that the key phosphoranyl radicals could be generated from phosphine oxides through a protonation/reduction sequence.

**FIGURE 14:**
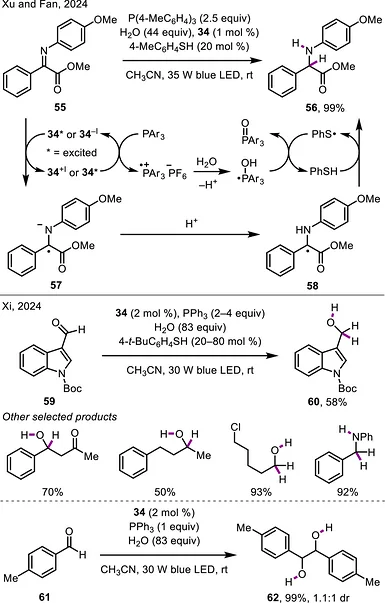
Hydrogenation of activated imines, aldehydes, and ketones with water‐derived phosphoranyl radicals under photocatalytic conditions.

Xu, Fan, and coworkers reported a photocatalytic protocol for imine reduction using phosphines and water (Figure 14) [63]. A single‐electron reduction of the imine substrate **55** by the excited state of the photoredox catalyst (34*) or its reduced ground‐state form (**34** ^−I^) was proposed to occur while the phosphine being oxidized to the corresponding radical cation intermediate. Subsequent protonation of the generated aza‐ketyl radical anion intermediate **57** to **58** followed by reduction with a thiol catalyst was suggested to afford **56** and a thiyl radical, which was proposed to be subsequently regenerated through a reduction with a water‐derived phosphoranyl radical. Chen and Xi achieved reductions of ketones, aldehydes, and aldimines to alcohols and amines, respectively, with water‐derived phosphoranyl radicals under photocatalytic conditions (see **59** to **60**, Figure 14) [64]. In addition, if a thiol HAT catalyst was excluded from the reaction mixture, pinacol‐type coupling products could be obtained (see **61** to **62**, Figure 14). In contrast to the report by Xu and Fan, a HAT transfer from a water‐derived phosphoranyl radical to closed‐shell substrates was suggested as the initial step of the reaction mechanism.

**FIGURE 15:**
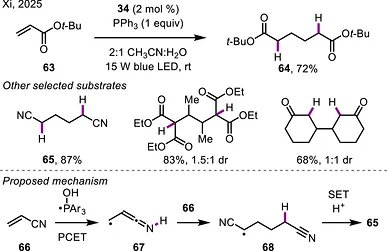
Reductive dimerization of activated electron‐deficient alkenes through PCET.

Xi and coworkers reported a formal reductive dimerization of activated electron‐deficient alkenes under photocatalytic conditions using water, phosphines, and an iridium(III) photoredox catalyst (see **63** to **64**, Figure 15) [65]. The water‐derived phosphoranyl radical was suggested to undergo a PCET to alkene substrate **66**, generating the corresponding allyl radical **67**. A Giese addition of **67** to another equivalent of substrate **66** was proposed to give the α‐cyano radical **68**, which underwent a SET reduction followed by protonation to eventually provide the dimeric product **65**. The authors carried out computational studies to rationalize the observed regioselectivity of the PCET process. Based on the study, they suggested that a hydrogen‐bonding interaction between the water‐derived phosphoranyl radical and the ester group steered the selectivity of the PCET process. In the absence of hydrogen‐bonding interactions, selective formation of an α‐ester radical via formal hydrogen‐atom addition at the β‐position was predicted through computations. This pathway would lead to a branched coupling product that was not experimentally detected.

**FIGURE 16:**
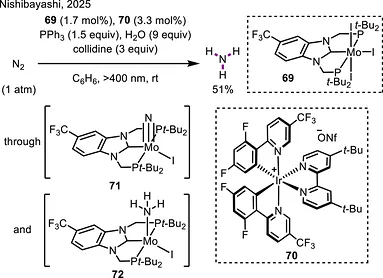
Mo‐catalyzed reduction of N_2_ to ammonia with water‐derived phosphoranyl radicals.

Nishibayashi employed water‐derived phosphoranyl radicals as a source of hydrogen atoms in visible‐light‐driven Mo‐catalyzed reductions of nitrogen to ammonia (Figure 16) [66]. Mechanistic studies suggested that the nucleophilic attack of water to triphenylphosphine radical cation was the rate‐determining step in ammonia generation. In agreement with this proposal, electron‐rich phosphine reagents were found to be less efficient in the developed process. The authors noted an inhibitory effect by phosphine oxide by‐products generated in stoichiometric quantities in the reaction; however, the specific inhibitory pathway was not identified. The reaction was proposed to proceed through activation of N_2_ by the Mo catalyst **69** to form molybdenum nitride complex **71**, which then underwent threefold PCET steps from phosphoranyl radicals to generate the ammonia complex **72**, thereby completing the reduction.

## 3 Weakening of Protic Bonds by Samarium(II) Complexes

In 1993, Hasegawa and Curran reported that water accelerated certain classes of reduction reactions promoted by SmI_2_ and proposed that water served not only as a proton source [67]. Kamochi and Kudo observed that SmI_2_ in the presence of water enabled reductions of carboxylic acids and their derivatives that were noted unreactive with SmI_2_ under anhydrous conditions [68, 69, 70]. Reductions of carboxylic acid derivatives with SmI_2_–H_2_O mixtures were later extensively studied also by Szostak and Procter [71, 72, 73, 74, 75]. In addition, Szostak, Spain, and Procter found that SmI_2_ in the presence of water promoted reductions of substrates with considerably higher reduction potentials than experimentally determined for SmI_2_(H_2_O)_n_ [76]. Consistent with these earlier reports, Chciuk, Anderson, and Flowers suggested that SmI_2_–water‐promoted reductions of certain substrates proceed through a PCET process from a water‐coordinated samarium complex rather than a stepwise electron transfer/proton transfer pathway [12, 77, 78, 79, 80]. They observed that the reduction of anthracene did not occur in the absence of water but was initiated with amounts of water lower than that required to increase the reducing power of SmI_2_, inconsistent with the reaction proceeding through an initial electron‐transfer step. In line with the proposed PCET mechanism, activation parameters pointing to a highly ordered early transition state and a primary isotope effect of 1.7 were determined in the reduction of anthracene. As the BDE of the carbon‐centered radical formed through the hydrogen‐atom transfer to anthracene is 44.9 kcal mol^−1^, coordination of water to SmI_2_ induced weakening of the O─H bond of water of at least 73 kcal mol^−1^. In a follow‐up study, Flowers and coworkers showed that only proton donors that coordinate to Sm^2+^ could participate in reductions of anthracene through a PCET process [81].

Flowers and coworkers also found that secondary amides could act as formal hydrogen‐atom donors in SmI_2_‐promoted reductions [82]. The use of SmI_2_ together with 2‐pyrrolidinone enabled reductions of anthracene, *trans*‐stilbene, and phenanthrene, which indicated weakening of the amide N─H bond upon coordination of about 71 kcal mol^−1^. Control experiments using *N*‐methyl‐2‐pyrrolidinone lacking the N─H bond or a combination of *N*‐methyl‐2‐pyrrolidinone and trifluoroethanol as noncoordinating proton source led to the complete recovery of the phenanthrene substrate, indicating that the coordination of the proton source was essential for the observed reactivity.

**FIGURE 17:**
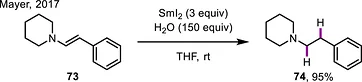
Enamine reduction with a Sm(II)–water system.

Kolmar and Mayer expanded the scope of reductions with the SmI_2_–water system through a PCET process to electron‐rich enamines (**73**), providing the corresponding amine products (**74**) (Figure 17) [83]. A thermochemical analysis of the developed system indicated that the O─H bond in water upon coordination to SmI_2_ has a BDFE of only 26 kcal mol^−1^. Despite the low BDFE value of the coordinated water, an undesired rapid evolution of H_2_ from aqueous solutions of SmI_2_ was not observed. The authors hypothesized that kinetic factors were likely responsible for the unexpected stability of the SmI_2_–water system.

Flowers and coworkers studied the effect of the nature of anionic halide ligands on the structure and reactivity of samarium(II)–water complexes [9, 84, 85]. Conductivity measurements and computational studies revealed that iodide ligands were fully displaced from SmI_2_ by water, forming cationic [Sm(H_2_O)_n_]^2+^2I^−^ complexes. Addition of water to SmBr_2_ led to only a partial dissociation of bromide ligands from Sm(II), reducing the number of water molecules that could coordinate to Sm(II). Chloride ligands of SmCl_2_ remained tightly bonded to Sm(II) upon addition of water, thus yielding a more electron‐rich and powerful reducing system as compared to SmI_2_ and SmBr_2_. Consistent with the structural effects of halide ligands on Sm(II)–water complexes, the degree of O─H‐bond weakening of water was estimated to be considerably larger in SmBr_2_–water (≥83 kcal mol^−1^) and SmCl_2_–water complexes (83–88.5 kcal mol^−1^) than in the system derived from SmI_2_ and water (73 kcal mol^−1^) [77]. Unfortunately, addition of water to SmCl_2_ led to an instantaneous generation of H_2_ gas, thus limiting the synthetic utility of this powerful reducing agent. By contrast, addition of 200 equivs of water to solutions of SmBr_2_ and SmI_2_ resulted in a much slower evolution of H_2_ over a period of 600–700 and 4000 s, respectively.

Flowers and coworkers studied the effect of the nature of the ligated protic molecule on the coordination‐induced‐bond‐weakening effect by SmI_2_ [86]. Ammonia was found to coordinate to samarium more strongly and, likely due to its bulkiness, to displace both iodide ligands to the secondary solvation shell of the Sm(II) complex. Both ammonia‐ and water‐derived Sm complexes were demonstrated to fully reduce acenaphthylene and anthracene but were found uncapable of reducing phenanthrene (C─H BDFE of the formal H‐atom adduct radical of phenanthrene is 25.3 kcal mol^−1^). The ammonia‐coordinated Sm(II) complex exhibited an order of magnitude greater rate of reduction of arene substrates than the corresponding complex with coordinated water. The BDFE value of the N─H bond of ammonia‐coordinated Sm(II) complexes was estimated as 34 kcal mol^−1^ (equals to 62 kcal mol^−1^ N─H bond weakening), which represents the largest degree of weakening of the N─H bond upon coordination reported to date.

Flowers and coworkers also compared the reactivity of Sm(II) complexes containing either coordinated water or methanol [87]. Coordination of methanol to Sm(II) resulted in O─H‐bond weakening of 52 kcal mol^−1^, substantially less than that previously determined for water [77]. Due to decreased coordination strength of methanol to Sm(II), reduction of substrates was proposed to proceed through a PCET process only at high concentrations of methanol. At low concentrations of methanol, a sequential electron–proton transfer to arene substrates was suggested.

**FIGURE 18:**
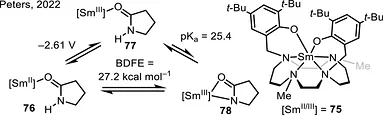
Determination of the H─N‐BDFE value in a Sm(II)‐2‐pyrrolidone complex.

Rigorous determination of thermodynamic parameters of samarium(II) complexes with coordinated protic ligands is often complicated by their poor solubility and ill‐defined speciation in solution. Boyd and Peters synthesized a Sm(II) complex containing a bulky macrocyclic ligand that provided a reliable system for assessing the BDFE values of coordinated protic ligands (Figure 18) [88]. The prepared complex **75** was evaluated in reductions of *trans*‐stilbene, 1,1‐diphenylethylene, and tetraphenylethylene, affording the corresponding products in 83%–92% yields. Determination of the reduction potential and p*K*_a_ values enabled to estimate the O─H BDFE value for the methanol‐coordinated complex as <24.1 kcal mol^−1^ and the N─H BDFE value of the 2‐pyrrolidone‐coordinated complex as 27.2 kcal mol^−1^, suggesting bond weakening of more than 72 and 69 kcal mol^−1^, respectively. The authors hypothesized that the main thermodynamic driving force for the bond weakening with samarium(II) was the high reductant strength of Sm(II) and the formation of the very strong Sm(III)–alkoxide or –pyrrolidonate bonds in the corresponding by‐products. It was shown that [Sm^III^]–2‐pyrrolidone **77** could be reversibly deprotonated by 1,8‐diazabicyclo[5.4.0]undec‐7‐ene (DBU) to **78** and reversibly electrochemically reduced to the Sm(II) state **76**, pointing to a possibility for regeneration of the PCET reductant and an opportunity for the design of Sm‐catalyzed systems.

**FIGURE 19:**
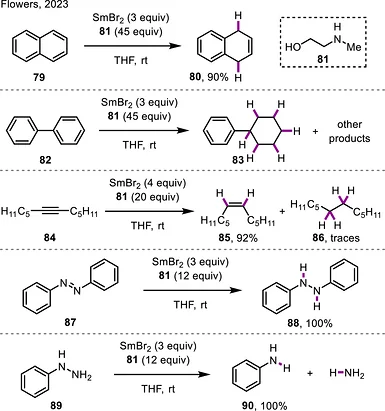
The use of the Sm(II)‐**81** complex in hydrogenation reactions of closed‐shell substrates.

Flowers and coworkers studied the effect of different chelating ligands on Sm(II)‐promoted PCET reactivity [89]. Most combinations between ethylene glycol or ethanolamine and Sm(II) complexes such as samarium dibromide, samarium dichloride, decamethyl samarocene, or samarium *bis*‐trimethylsilyl amide led to a rapid evolution of hydrogen gas, thus limiting their synthetic applicability. Further optimization studies identified SmBr_2_ in combination with *N*‐methylethanolamine (**81**) as the most stable complex capable of PCET reactivity. Reductions of *trans*‐stilbene and naphthalene (**79**) proceeded cleanly with SmBr_2_ and **81** to produce 1,2‐diphenylethane and 1,4‐dihydronaphthalene (**80**), respectively, while the reduction of biphenyl (**82**) yielded multiple products including cyclohexylbenzene (**83**, Figure 19). The developed system was found to be efficient in partial hydrogenations of alkynes (**84**) to *cis*‐alkene products (**85**) along with small amounts of the overhydrogenated alkanes (**86**). The Sm–**81** system was found to enable smooth reductions of unactivated esters and five‐to‐seven‐membered lactones. Reduction of ester substrates carrying an unactivated olefin afforded five‐membered cyclic alcohols as complex diastereomeric mixtures through 5‐*exo*‐trig radical cyclization of the initially generated ketyl‐type radical. Finally, reductions of azobenzene (**87**) and phenyl‐hydrazine (**89**) provided the corresponding products **88**, **90**, and ammonia in quantitative yields. As substrate reductions were also observed with *N*,*N*‐dimethylethanolamine but not with ethylenediamine, the authors suggested that the hydrogen atom originated from the O─H bond of **81** rather than from the N─H bond.

**FIGURE 20:**
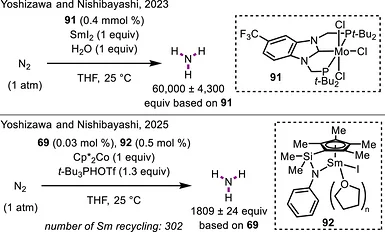
Mo‐catalyzed reduction of N_2_ to ammonia using Sm(II)‐water complexes.

Nishibayashi and coworkers studied the application of the SmI_2_–water system in the presence of molybdenum‐derived catalysts for reduction of N_2_ to ammonia (Figure 20) [90, 91]. The most effective molybdenum trichloride catalyst **91** bearing a trifluoromethyl‐substituted PCP‐type pincer ligand was demonstrated to produce up to 60 000 equivalents of ammonia based on the molybdenum catalyst (79% based on the Sm(II) reductant), with a catalytic turnover frequency of 800 equiv min^−1^. Study of the kinetic profile of the system revealed that the rate‐determining step differed based on the catalyst concentration. At high catalyst concentrations, the PCET step resulting in the formation of the N─H bond was the slowest step of the sequence, while dimerization of the catalyst was found to be the rate‐determining step at low catalyst concentrations.

In 2025, Nishibayashi and coworkers further elaborated their N_2_‐reduction protocol and reported a system that was catalytic in both the samarium reductant and the molybdenum complex [92]. Cyclopentadienyl–amide chelate ligands on samarium (see **92**) were suggested to prevent polynucleation of Sm species. The Sm‐complexes were recycled over 300 times in the developed reaction system and were found to increase the rate of ammonia formation seven times as compared to the rate in the absence of a Sm‐complex. In situ protonation with a phosphonium‐salt acid and reduction with bis(pentamethylcyclopentadienyl)cobalt(II) enabled the efficient recycling of the PCET‐active samarium(II) complex. Along the same lines, Peters and coworkers developed a catalytic system for reduction of N_2_ to ammonia [93]. Samarium(II) reductants were regenerated from oxidized Sm(III) by‐products electrochemically in the presence of a 2,6‐dimethylpyridinium bis(trifluoromethanesulfonamide) salt as the proton source in the absence of alcohol or water additives.

## 4 Weakening of Protic Bonds by Complexes Containing Redox‐Active Metal Atoms

### 4.1 Manganese

**FIGURE 21:**
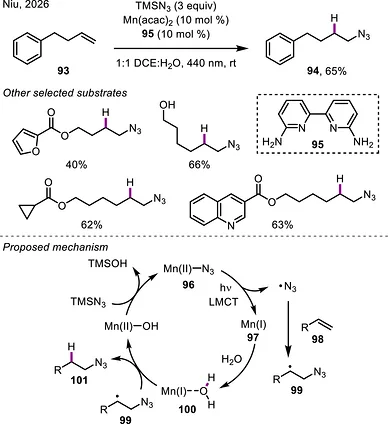
O─H‐Bond weakening of water with manganese complexes.

Niu and coworkers developed a manganese‐catalyzed anti‐Markovnikov hydroazidation of alkenes that included water as the source of hydrogen atom (Figure 21) [94]. A variety of unactivated alkene substrates (e.g., **93**) underwent hydro‐functionalization reaction producing the corresponding alkyl azide products (**94**). Substrates containing sensitive functional groups such as electron‐rich heterocycles, free alcohols, strained rings, or basic groups were found to be compatible with the developed conditions. In situ generated manganese(II)–azide complex **96** was proposed to undergo light‐induced homolysis releasing an azidyl radical and the corresponding manganese(I) complex **97**. Addition of the azidyl radical to alkene substrate **98** followed by hydrogen‐atom transfer from the putative water‐coordinated manganese(I) complex **100** to alkyl radical **99** was suggested to provide the hydro‐functionalized product **101** while re‐oxidizing the manganese complex to its initial oxidation state.

### 4.2 Iron

**FIGURE 22:**
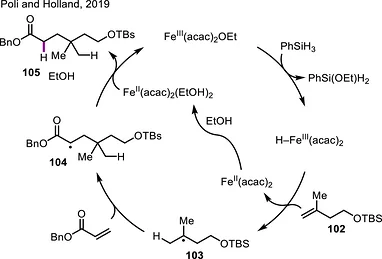
Revised mechanism of Fe‐catalyzed reductive alkene/alkene cross‐coupling reactions.

Poli, Holland, and coworkers studied the mechanism of iron‐catalyzed radical alkene cross‐coupling reactions that was originally proposed to include the conversion of an α‐ester radical to the corresponding product through an electron‐transfer/proton‐transfer sequence [95, 96]. In the overall reaction, the (acac)_2_Fe(III)–H complex was proposed to first undergo a hydrogen‐atom transfer with electron rich alkene **102** to generate tertiary alkyl radical **103** (Figure 22). The tertiary radical **103** was suggested to further react in a Giese addition with electron‐poor *tert*‐butyl acrylate to give the corresponding α‐ester radical **104**. Poli, Holland, and coworkers found that the iron(II) complex showed too little reducing power to enable a direct electron transfer to the α‐carbonyl radical **104**, and thus the originally proposed stepwise electron‐transfer/proton‐transfer mechanism was regarded as unlikely. Computational analysis revealed that the electron transfer would be endothermic by 35 kcal mol^−1^ and would require overcoming a large reorganization energy barrier of 26 kcal mol^−1^. Holland and coworkers alternatively proposed that a PCET from an iron(II)–ethanol complex as a more plausible mechanism for the formation of the final product **105** along with the (acac)_2_Fe(III)–OEt complex. Computational studies revealed that coordination of ethanol to Fe(acac)_2_ lowered BDFE of EtO─H from 104 to 74 kcal mol^−1^, consistent with their conclusion. In addition, the authors experimentally demonstrated that an iron(II)–ethanol complex could reduce a cyanoisopropyl carbon‐centered radical derived from azobis(isobutyronitrile) (AIBN), yielding the corresponding product featuring a newly formed C─H bond of 92 kcal mol^−1^. The Fe‐catalytic cycle is completed by ligand exchange of the (acac)_2_Fe(III)–OEt complex with phenyl silane to regenerate the initial Fe(III)─H complex.

**FIGURE 23:**
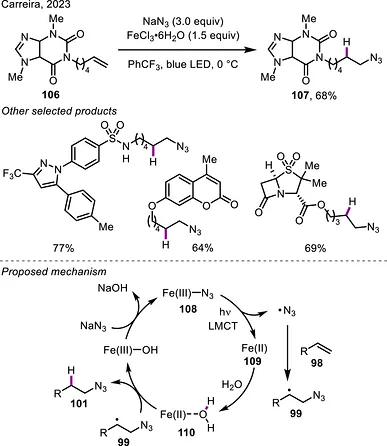
Fe‐catalyzed radical *anti*‐Markovnikov hydroazidation.

Carreira and coworkers developed an iron‐catalyzed *anti*‐Markovnikov hydroazidation of unactivated alkenes, in which water was found to serve as the source of hydrogen atoms [97]. For example, the theobromine derivative **106** was regioselectively hydroazidated with NaN_3_ to terminal alkyl azide **107** (Figure 23). The azidyl radical was proposed to arise from the corresponding Fe(III)–azido complex **108** through a photomediated ligand‐to‐metal‐charge transfer (LMCT). Regioselective addition of the azidyl radical to alkene **98** provides the β‐azido alkyl radical **99**. The subsequent hydrogen‐atom transfer to **99** was hypothesized to occur from the putative Fe(II)–water complex **110**, yielding the hydroazidated product **101** along with the corresponding Fe(III)–hydroxo complex. Ligand exchange with an azide anion was proposed to regenerate **108**, thus closing the catalytic cycle.

**FIGURE 24:**
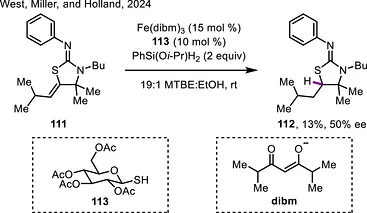
Enantioselective Fe‐catalyzed hydrogenation through a proposed Fe(II)–thiol complex.

West, Miller, Holland, and coworkers developed an enantioselective Fe‐catalyzed hydrogenation of olefins, providing a variety of enantioenriched cyclic ureas and thiazolidines (e.g., **112**, Figure 24) [98]. Mechanistic analysis of the developed system was consistent with an enantiodetermining PCET from a chiral iron(II)–thiol complex to a carbon‐centered radical intermediate generated from **111** by an iron(III)–hydride complex. DFT analysis of the catalytic system suggested weakening of the thiol S─H bond from 82 to 66 kcal mol^−1^ upon coordination to Fe(II).

**FIGURE 25:**
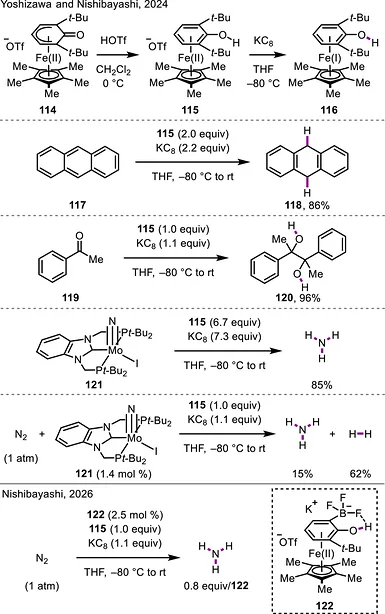
Radical hydrogenations with Fe(I)‐complexes bearing protic π‐phenol ligands.

Yoshizawa and Nishibayashi reported an iron(I) complex containing η^6^‐di‐*tert*‐butylphenol ligand (**116**) with the phenolic O─H bond with BDFE of 27.8 kcal mol^−1^(Figure 25) [99]. The active iron(I) complex **116** was prepared from the corresponding iron(II) complex **114** by protonation with triflic acid, yielding complex **115**, and in‐situ reduction with KC_8_. Complex **116** enabled PCET to a variety of closed‐shell substrates. For example, anthracene (**117**) was converted to 9,10‐dihydroantharcene (**118**) in 86% yield besides 13% of dihydrogen as a side‐product. Reduction of acetophenone (**119**) through formation of ketyl radical intermediates with O─H BDFE values of 39 kcal mol^−1^ provided the corresponding pinacol coupling product **120** in 96% yield. Reduction of the molybdenum nitride complex **121** derived from dinitrogen provided 85% yield of ammonia based on the amount of the molybdenum complex. The authors also investigated the catalytic version of nitrogen reduction using a molybdenum catalytic system. A stoichiometric amount of the active iron complex generated in situ in the presence of 1.4 mol % of **121** under atmospheric pressure of nitrogen yielded 15% of ammonia. The process also produced 62% yield of dihydrogen side‐product, suggesting that a low catalytic activity of the molybdenum catalyst was responsible for the poor yield of ammonia formation.

In a follow‐up study, the Nishibayashi group showed that the reactivity of the iron–phenol complex could be tuned by ligand design [100]. A ligand containing a trifluoroborate substituent was designed that was proposed to further stabilize the phenolic O─H bond through hydrogen bonding. Indeed, the strength of the phenolic O─H bond was determined as 40.7 kcal mol^−1^, and the complex was found to be more resistant against a spontaneous dihydrogen evolution. The developed complex **122** was successfully applied to the hydrogenation of anthracene and the reductive coupling of acetophenone. Reduction of a molybdenum‐nitride complex with the iron(I) complex afforded 68% of ammonia based on the amount of the molybdenum complex. The authors also attempted to use the prepared complex **122** in a catalytic dinitrogen reduction. However, only 0.8 equiv of ammonia was generated based on the amount of the molybdenum catalyst used. The authors hypothesized that the reason for the low efficiency of the process was the high BDFE of the prepared complex compared to the newly formed N─H bond (40.7 kcal mol^−1^ vs. 33.8 kcal mol^−1^), in addition to a slow N─N bond cleavage and ammonia‐to‐dinitrogen ligand exchange under the low reaction temperature that was required for the stability of the iron(I) complex.

**FIGURE 26:**
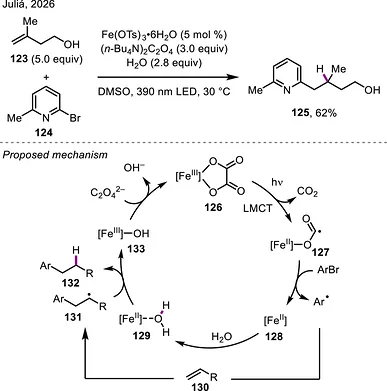
Fe‐catalyzed radical hydroarylation with water as the formal hydrogen‐atom donor.

Coordination‐induced bond weakening of water was also proposed as a possible mechanism, in addition to a radical‐polar crossover pathway, for hydrogen‐atom transfer in alkene hydroarylations promoted by ferrioxalate photocatalysis developed by Juliá and coworkers (see **123** and **124** converted to **125**, Figure 26) [101]. The proposed mechanism includes irradiation of ferrioxalate (**126**) that triggers release of CO_2_, resulting in the formation of Fe(II)oxycarbonyl radical **127**. Radical **127** engages as a strong reductant in the SET‐reduction of an aryl bromide to generate the corresponding aryl radical along with Fe(II)‐complex **128** and another equivalent of CO_2_. The aryl radical in turn adds to alkene **130** to generate β‐arylalkyl radical **131** that is eventually reduced by Fe(II)–water complex **129** to afford the hydroarylation product **132** along with Fe(III)‐OH complex **133**. Consistent with the proposed mechanism, the use of D_2_O instead of H_2_O provided the corresponding products with <60% deuterium incorporation.

### 4.3 Titanium

Oloyede and Flowers recently presented a comprehensive perspective on the coordination‐induced bond weakening phenomenon in low‐valent titanium complexes [4]. Therefore, this section will highlight only a few selected examples that illustrate the synthetic utility of titanium complexes in formal hydrogen‐atom transfer reactions from protic molecules.

**FIGURE 27:**
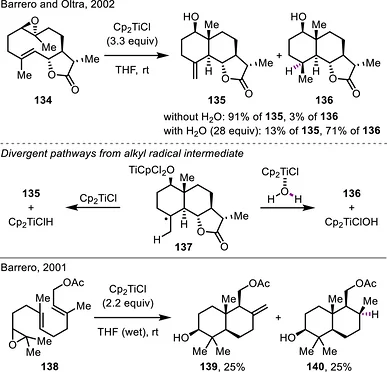
Ti(III)‐mediated cascade reductive epoxide opening. In the presence of water, reduction of cyclized tertiary C‐radical intermediates was achieved.

In the course of synthetic efforts toward natural eudesmanolides, Barrero, Oltra, and coworkers discovered that addition of water to a transannular cyclization of epoxide **134** mediated by Cp_2_TiCl resulted in the formation of reduced product **136** as the major compound (71% yield) instead of the expected exocyclic alkene **135** (Figure 27) [102, 103]. By contrast, alkene **135** could be obtained in 91% yield by conducting the reaction under anhydrous conditions. Experiments using D_2_O yielded the corresponding deuterated product, consistent with the hypothesis that water was the source of hydrogen atoms. The authors proposed that coordination of water to a titanium(III) complex enabled water to provide hydrogen‐atom equivalents for reduction of the cyclized tertiary alkyl radical intermediate **137**. A similar observation was reported in a titanium(III)‐mediated cascade cyclization of epoxypolyprene **138**, which afforded a 1:1 mixture alkene **139** and alkane **140** when conducted in a wet THF solvent. By contrast, the use of strictly anhydrous reaction conditions allowed formation of **139** in 40% yield while suppressing the pathway leading to the undesired reduced alkane product **140** (Figure 27) [104, 105].

**FIGURE 28:**
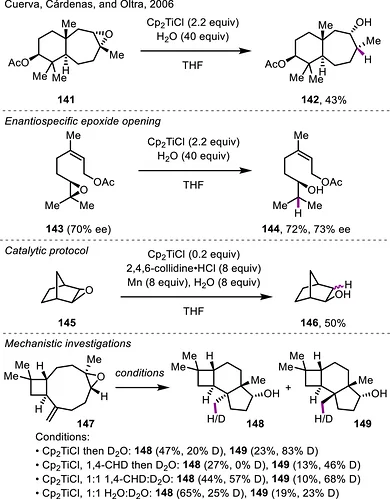
Ti(III)‐mediated reductive epoxide opening and related mechanistic studies.

Cuerva, Cárdenas, Oltra, and coworkers expanded the substrate scope of the reductive epoxide opening with water and Cp_2_TiCl and showed that high regio‐ and stereoselectivity could be achieved, for example, in conversion of epoxide **141** to alcohol **142** (Figure 28) [106]. In addition, an excellent enantiospecificity was observed in reduction of enantioenriched epoxide **143** to alcohol **144**. Finally, a catalytic variant of the reductive epoxide opening was developed using 20 mol % of Cp_2_TiCl, which afforded comparable yields of the corresponding alcohol products (**146** obtained from **145** in 50% yield under catalytic conditions while 60% yield of **146** was obtained under the original stoichiometric conditions). Superstoichiometric quantities of 2,4,6‐collidine hydrochloride and elemental manganese were used as the titanocene‐regenerating agents under the catalytic conditions.

The authors also provided strong evidence that water served as the hydrogen‐atom source in the reductions of alkyl radicals in the presence of titanium(III) complexes [106]. They observed that in reductions of epoxide **147**, a deuterium‐atom transfer from a D_2_O‐coordinated Ti(III)‐complex was faster than a hydrogen‐atom transfer from cyclohexa‐1,4‐diene (1,4‐CHD), which in turn was more rapid than the formation of an alkyl–Ti(IV) complex followed by hydrolysis (see products **148** and **149**, Figure 28). DFT analysis of the proposed titanocene–water complex revealed that the O─H bond of water was weakened by 58.7 kcal mol^−1^ upon coordination. An analogous analysis of the zirconocene‐ and hafnocene‐water complexes demonstrated coordination‐induced O─H‐bond weakening by 82.6 and 94.4 kcal mol^−1^, respectively, suggesting that also other metals capable of single‐electron transfer might enable water to act as a source of hydrogen atom.

Titanium(III) complexes bearing a variety of protic ligands were studied by Cárdenas, Oltra, Cuerva, and co‐workers [107]. Evaluated ligands showed smaller bond weakening upon coordination to titanocene than water (e.g., MeOH: 50.9 kcal mol^−1^; PhOH: 34.7 kcal mol^−1^; NH_3_: 35.4 kcal mol^−1^; Me_2_NH: 19.1 kcal mol^−1^). The authors concluded that water was an excellent hydrogen‐atom donor upon coordination to titanium(III) owing to its strong binding affinity and the low activation energy of the hydrogen‐atom‐transfer step (corresponding to O─H‐bond weakening of 58.6 kcal mol^−1^). Along these lines, Jin and Newcomb experimentally determined the rate constant for the hydrogen‐atom transfer from the titanocene–water complex to a secondary alkyl radical in THF at ambient temperature as 1.0 × 10^5^ M^−1^ s^−1^ using radical‐clock experiments [108]. Gansäuer, Friedrich, van Gastel, and coworkers studied the structure of the water‐coordinated Ti(III)‐complex by spectroscopic, electrochemical, and computational methods and proposed that the active HAT reagent was a cationic Ti(III) complex rather than a neutral species [109].

**FIGURE 29:**
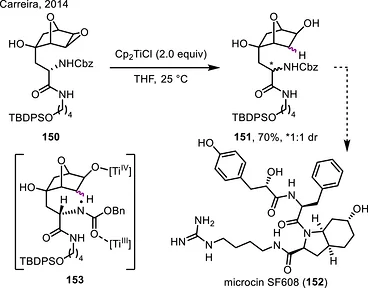
Regioselective Ti(III)‐mediated epoxide reduction through an intramolecular hydrogen‐atom delivery.

In the course of synthetic studies toward the serine protease inhibitor microcin SF608 (**152**), Carreira and coworkers observed that a titanium(III)‐promoted reduction of epoxide **150** delivered alcohol **151** with unexpectedly high regioselectivity (Figure 29) [110]. Mechanistic studies suggested that the high regioselectivity was enabled by an intramolecular hydrogen‐atom delivery from the adjacent carbamate NH group activated by coordination to Cp_2_TiCl, thus selectively reducing one of the two rapidly interconverting β‐Ti(IV)oxy‐alkyl radical regioisomers. A complete epimerization of the α‐amino stereocenter was observed in the process, which was rationalized by a formal hydrogen‐atom migration to the nitrogen‐centered radical (**153**) yielding a more stable carbon‐centered radical.

**FIGURE 30:**
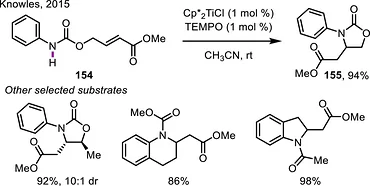
Cyclization of carbamates and amides through Ti catalysis with the TEMPO radical as a catalytic HAT reagent.

Knowles and coworkers developed a cocatalytic system for conjugate aminations, in which coordination of *N*‐aryl amide substrates (**154**) to reducing titanocene(III) complexes enabled a formal abstraction of hydrogen atoms with TEMPO radicals through a PCET process [111]. Addition of the resulting titanium(IV)‐aza‐enolate intermediates to electron‐deficient olefins, followed by metal migration, and proton‐ and electron‐transfer steps provided the aminated products (**155**, Figure 30). Undesired radical coupling between the titanocene and TEMPO catalysts leading to their deactivation was efficiently suppressed by using bulky Cp* ligands associated with the titanocene catalyst. DFT analysis of the cocatalytic system revealed that coordination of the amide substrate to titanium(III) weakened the N─H bond by 33 kcal mol^−1^, thereby rendering the subsequent formal hydrogen‐atom abstraction by TEMPO nearly thermoneutral.

**FIGURE 31:**
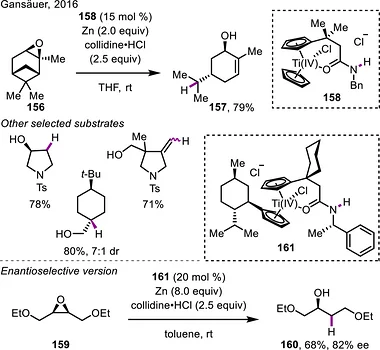
Bifunctional Ti‐complexes as catalysts in reductive opening of epoxides.

Gansäuer and coworkers developed titanocene(III) complex generated in situ from **158** bearing a pendant NH amide group, in which the SET reductant and the protic donor were combined within a single species (Figure 31) [112]. The bifunctional Ti(III)‐catalyst was demonstrated to efficiently mediate both steps in reductions of epoxide substrates (**156**), the epoxide opening and the subsequent hydrogen‐atom transfer to yield the corresponding alcohol products (**157**). The developed reaction required the use of stoichiometric amounts of zinc reductant and collidine•HCl complex as a proton source. Following this strategy, chiral Ti(III)‐catalysts containing enantiopure menthyl‐derived cyclopentadienyl ligands were prepared. Reduction of symmetric epoxide **159** using catalyst **161** provided alcohol **160** in a good yield and 82% ee (Figure 31). In addition, a cocatalytic system was developed that included **161** and Crabtree's hydrogenation catalyst [Ir(cod)PyPCy_3_]PF_6_, which enabled lowering the reaction temperature to 10 °C, thus achieving higher enantioselectivity in this transformation (91% ee). The amide‐titanocene complex was proposed to catalyze the epoxide opening and act as a hydrogenating agent in the cocatalytic system to provide the corresponding iridium hydride complexes.

**FIGURE 32:**
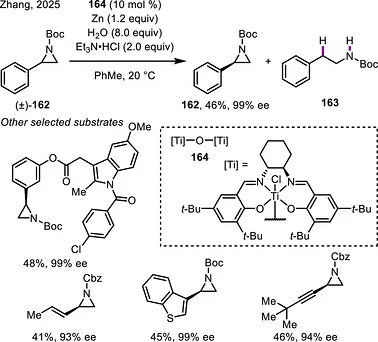
Kinetic resolution of aziridines through reductive ring opening with chiral Ti(III)‐complexes.

Zhang and coworkers developed a protocol for radical kinetic resolution of terminal activated *N*‐carbamate aziridines using titanium catalysis and water as a hydrogen‐atom source (Figure 32) [113]. The titanium catalyst was proposed to have a dual role in the system, enabling the enantioselective aziridine reductive opening and water activation for a hydrogen‐atom transfer to the resulting alkyl radicals. A chiral dinuclear salen titanium complex **164** in combination with water and zinc as a reductant was identified as the optimal reaction system for obtaining the model aziridine **162** in 46% yield and 99% ee with the corresponding amine **163** formed in 50% yield. A control experiment conducted in the absence of water ruled out the possibility that the ring‐opened product **163** formed through a single‐electron reduction of the alkyl radical intermediate followed by protonation, as only trace amounts of **163** were detected under the anhydrous conditions. In addition, DFT calculations of the titanium(III)–water complex revealed a coordination‐induced bond weakening of the O─H bond of water by 69 kcal mol^−1^.

In addition to the reductive opening of epoxides, the combination of Cp_2_TiCl (generated in situ from Cp_2_TiCl_2_ and Zn) and water was found to reduce ketones to the corresponding secondary alcohol products [114]. In the original study, the role of water was suggested as a proton source. However, mechanistic studies reported in 2010 revealed that titanium(III)‐promoted ketone reductions in aqueous media likely proceeded through reductions of the initially formed Ti(IV)‐ketyl radicals by water‐coordinated titanium(III) complexes rather than through a sequential electron‐transfer/proton‐transfer process [115].

**FIGURE 33:**
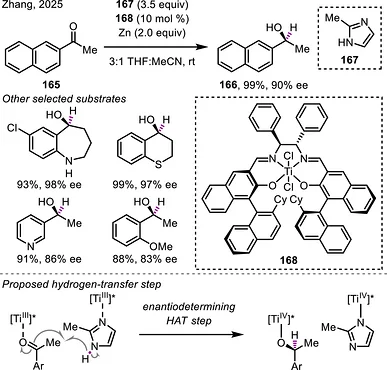
Enantioselective reductions of ketones using chiral Ti(III)‐complex **168** and imidazole **167**.

Zhang and coworkers developed a cooperative bimetallic catalytic system that enabled enantioselective reductions of carbonyl substrates via hydrogen‐atom transfer [116]. For example, methyl naphthyl ketone (**165**) was quantitatively reduced to secondary alcohol **166** in 90% ee (Figure 33). The method was found to tolerate sensitive functional groups such as boronate esters, electron‐rich and electron‐poor heterocycles, while providing the corresponding alcohol products with excellent enantioselectivity. The observed second‐order dependence on titanium catalyst **168** and the positive nonlinear‐effect correlation between the enantiomeric composition of **168** and the product enantioselectivity was consistent with two molecules of **168** cooperatively activating carbonyl acceptors and the imidazole hydrogen‐atom donor **167** (Figure 33). Radical‐clock experiments pointed to a concerted HAT process without involvement of carbon‐centered radical intermediates as the major reaction pathway. Computational analysis of the (salen)titanium(III)‐imidazole complex revealed weakening of the N─H bond of imidazole of 29 kcal mol^−1^ upon coordination.

**FIGURE 34:**
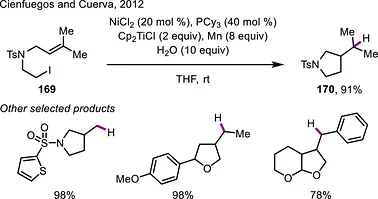
Reductive cyclization of alkyl iodides.

A formal hydrogen‐atom transfer from a titanium(III)–water complex was also proposed operative in nickel‐catalyzed Heck‐type radical cyclizations of alkyl iodides (**169**) to five‐membered products (**170**) (Figure 34) [117]. The nickel catalyst was proposed to generate alkyl radicals from the alkyl iodide substrates through a Ni(I)/Ni(II) catalytic cycle, which were reduced by the putative titanocene(III)–water complex upon cyclization.

**FIGURE 35:**
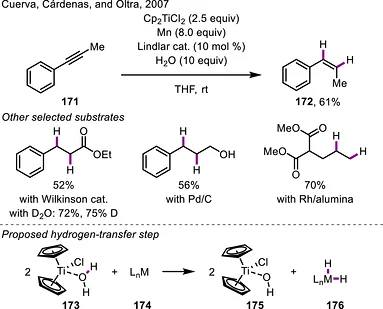
Application of titanocene–water complex in transition‐metal‐catalyzed hydrogenation reactions.

Cuerva, Cárdenas, Oltra, and coworkers applied the titanocene(III)–water system in transition‐metal‐catalyzed hydrogenations of alkenes and alkynes using a variety of common hydrogenation catalysts [118]. For example, alkyne **171** was semihydrogenated using the Lindlar catalyst to yield **172** in 61% yield (Figure 35). The use of D_2_O instead of water enabled bisdeuteration of ethyl cinnamate to give the corresponding product in 72% yield and 75% deuterium incorporation using Wilkinson's catalyst. The titanocene‐water complex **173** was observed to be stable under oxygen‐free conditions, which suggested that the catalyst did not act as a source of H_2_ under the developed conditions. The authors proposed that **173** promoted the hydrogenation reaction by hydrogen‐atom transfer to the hydrogenation catalysts **174** to give the active L_n_MH_2_ **176** together with the Cp_2_TiClOH by‐product (**175**).

### 4.4 Molybdenum

**FIGURE 36:**
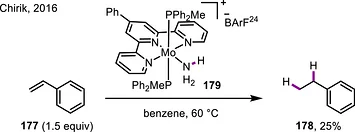
Ammonia N─H‐bond weakening in hydrogenation of styrene.

Chirik and coworkers reported a molybdenum complex capable of activating ammonia, water, and hydrazine X─H bonds [119]. The ammonia‐coordinated complex **179** was shown to transfer a hydrogen atom to 2,4,6‐tri‐*tert*‐butylphenoxyl radical (BDFE of 77 kcal mol^−1^) and to [(η^5^‐C_5_Me_5_)Cr(CO)_3_]_2_ (BDFE of 62 kcal mol^−1^). In addition, complex **179** enabled dihydrogen evolution at 60 °C and hydrogenation of styrene (**177**) in 25% yield (**178**, Figure 36). DFT calculations and experimental studies determined the BDFE of the ammonia‐coordinated complex of 45 kcal mol^−1^. In addition, molybdenum‐derived complexes with coordinated water and hydrazine were prepared. The corresponding X─H bond strengths were calculated as 34 kcal mol^−1^ for the O─H bond and 35 kcal mol^−1^ for the N─H bond.

### 4.5 Cobalt

In 2020, Peters and coworkers disclosed cobaltocenium–aniline complex **180** that mediated PCET reactivity [120]. Attachment of the basic aniline group to cobaltocene facilitated protonation of the complex, which was together with a single‐electron reduction necessary for the subsequent PCET reactivity. In addition, the spatial separation of the redox‐active cobaltocenium and the proton source suppressed the undesired H_2_ evolution pathway, observed previously with the simple protonated cobaltocenium (Cp_2_Co^+^) PCET reagent. In addition, the authors proposed that a pathway toward H_2_ evolution would require coupling of two positively charged cobaltocenium species, which was suggested as unfavorable. The BDFE of the N─H bond of cobalt(III) complex **180** upon *N*‐protonation was experimentally determined as 79 kcal mol^−1^. Single‐electron reduction yielding cobaltocene(II)–anilinium complex lowered the strength of the N─H bond by 40 kcal mol^−1^.

**FIGURE 37:**
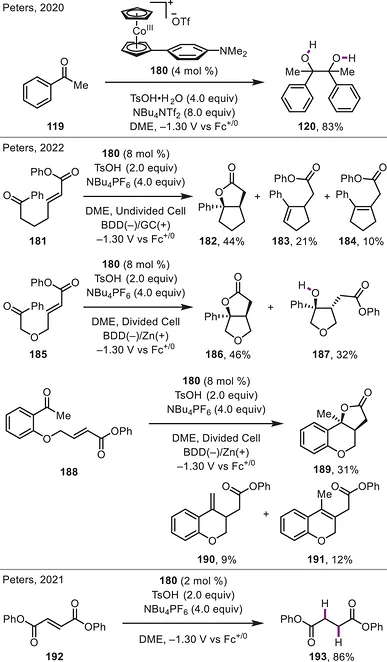
Reductive dimerization of acetophenone, intramolecular ketone/olefin couplings, and hydrogenation through PCET using cobalt‐derived complex **180**.

Generation of ketyl radicals from carbonyl compounds requires relatively low reduction potentials (less than −2.0 V vs Fc^+/0^), which limits their synthetic utility [121]. By contrast, the electrochemically promoted PCET reactivity of cobaltocenium(II)–anilinium complex under mildly acidic conditions enabled controlled‐potential coulometry experiments at −1.30 V versus Fc^+/0^ that yielded the corresponding pinacol coupling adduct of acetophenone **120** in 83% yield, together with 11% of the recovered acetophenone (**119**, Figure 37). Kinetic isotope effect study suggested a rate‐determining reductive PCET to form the neutral ketyl radical of acetophenone. A Hammett analysis of reductions of *para*‐substituted acetophenone substrates revealed a slight buildup of positive charge on the acetophenone substrate in the transition state, consistent with an asynchronous PCET reaction.

Subsequent studies by Peters and coworkers extended the application of their system to reductive ketyl–olefin cyclization providing cyclic alcohol products (Figure 37) [121]. Note that the initial ketyl–olefin cyclization product derived from **181** was not stable under the reaction conditions and engaged in lactonization to afford the major product **182** and the isomeric water‐elimination products **183** and **184**. Control experiments demonstrated the essential role of the PCET mediator, as no products were generated in the absence of **180** at −1.30 V versus Fc^+/0^. Hammet analysis of the reaction with 4‐substituted aryl keto–olefin substrates revealed an inverse relationship between the rate and the Hammett parameters, in an analogous way as observed for acetophenone reduction [120], consistent with the reduction of the carbonyl group to a ketyl radical being the initial and rate‐determining step. The mild conditions allowed cyclizations of substrates containing sensitive groups such as halide substituents or heterocycles. Further optimization of the reaction setup and conditions afforded access to pyrrolidine and tetrahydrofuran derivatives (see **185** to **186** and **187**). In addition, a 6‐*exo*‐trig cyclization was achieved with substrate **188** yielding the corresponding chromane products **189**, **190**, and **191** (Figure 37).

Peters and coworkers expanded the scope of substrates of PCET reductions with **180** to hydrogenations of C─C π‐systems [122]. Reduction of fumarate esters was proposed to be thermodynamically feasible as the BDFE of the newly formed C─H bond in the resulting succinyl radical intermediate was calculated by DFT as 48 kcal mol^−1^. Consistent with this consideration, diphenyl succinate (**193**) was generated by controlled‐potential coulometry in an undivided cell from diphenyl fumarate (**192**) in 86% yield (Figure 37). Control experiments supported the key role of **180**, as product **193** was obtained only in 24% yield in the absence of **180** and in 9% yield when using Cp_2_Co instead of **180**.

**FIGURE 38:**
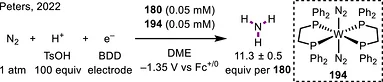
Electrochemical reduction of N_2_ using tungsten–(N_2_)_2_ complex **194** and cobalt‐derived PCET mediator **180**.

A cocatalytic reduction of N_2_ to ammonia at applied potential of −1.2 V versus Fc^+/0^ was achieved by Peters and coworkers using the developed PCET system [123]. As the PCET mediator was not capable of reducing N_2_ directly, a tandem cocatalytic strategy was devised that included a molecular catalyst able to bind N_2_ and facilitate its multistep reduction through multiple reactive intermediates. The authors adopted a system initially studied by Pickett, which relied on a tungsten‐derived complex [124]. Controlled‐potential coulometry under the original conditions by Pickett using tungsten–(N_2_)_2_ complex **194** and the cobalt PCET mediator **180** at −1.35 V versus Fc^+/0^ delivered 4.7 ± 0.3 equivs of ammonia. Optimization of the reaction conditions enabled generation of 11.3 ± 0.5 equivs of ammonia (Figure 38). Other metal complexes capable of activating N_2_ based on Os, Fe, or Mo were found to also mediate the N_2_ reduction in the presence of **180**.

**FIGURE 39:**
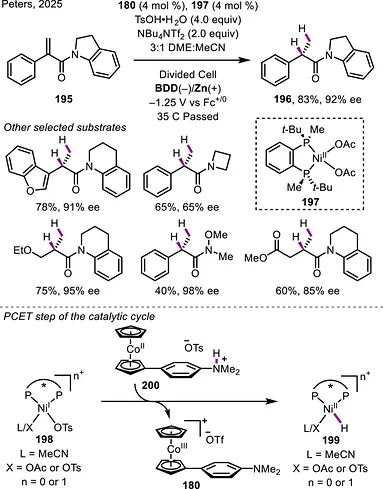
Enantioselective electrochemical reduction of α,β‐unsaturated amides using the cobaltocene‐derived PCET mediator **180** in cooperation with Ni‐catalyst **197**.

The cobaltocene‐derived PCET mediator **180** was also applied in enantioselective hydrogenations of unsaturated alkenes using chiral nickel cocatalyst **197** under electrocatalytic conditions (Figure 39) [125]. The PCET mediator **180** was proposed to deliver a hydrogen atom to the nickel hydrogenation catalyst **198**, forming the Ni^II^─H complex **199** suitable for substrate reduction. With this method, α,β‐unsaturated amide **195** was successfully hydrogenated to give **196** in high yield and excellent enantioselectivity.

## 5 Weakening of Protic Bonds by Heterobimetallic Complexes or Complexes Containing Redox‐Active Ligands

### 5.1 Aluminum

**FIGURE 40:**
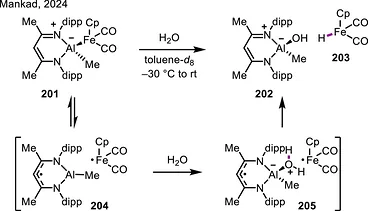
Redox‐active ligands associated with redox‐inactive aluminum(III) can induce O─H‐bond weakening of water.

Aluminum(III) is a redox‐inactive metal under mild conditions, and therefore, cannot alone induce bond weakening of protic ligands. Mankad and coworkers developed a system enabling large levels of coordination‐induced weakening of O─H bonds of water and alcohols, and N─H bonds of amines by employing a redox‐active ligand associated with aluminum(III) central atom (**201**, Figure 40) through MS‐PCET mechanism [126]. Complex **201** was proposed to dissociate reversibly by homolysis of the Al─Fe bond, thus generating a small concentration of frustrated radical pair **204**. The resulting frustrated radical pair was shown to react with water, simple alcohols, and amines to give [Al─X] (**202**) and [H─Fe] (**203**) complexes. DFT analysis of the aluminum(III)–water complex **205** revealed a considerable bond‐weakening effect induced by the aluminum complex, with the BDFE of the O─H bond of the coordinated water determined as only 1.0 kcal mol^−1^.

### 5.2 Vanadium

**FIGURE 41:**
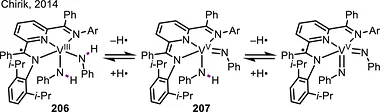
Formal hydrogen‐atom transfers from aniline N─H‐bonds through activation with vanadium complexes containing redox‐active ligands.

Vanadium complexes containing redox‐active pyridine diimine ligands were reported by Chirik and coworkers [127]. The BDFE of the N─H bonds of the prepared complexes was found to be influenced by the oxidation state of both the metal center and the bis(imino)pyridine ligand. Vanadium(V) complex **207** containing a two‐electron reduced chelate was calculated to contain N─H bond with BDFE of 69.2 kcal mol^−1^, while the vanadium(III) complex **206** with a one‐electron reduced chelating ligand was reported to have weaker N─H bonds of BDFE of 64.1 kcal mol^−1^ (Figure 41).

**FIGURE 42:**
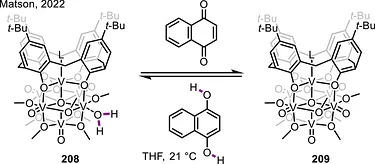
O─H‐bond weakening of water by polyoxovanadate cluster.

Matson and coworkers prepared polyoxovanadate cluster **208** containing aqua ligands as a model for studying heterogeneous metal‐oxide material surfaces (Figure 42) [128]. Experimental equilibrium studies indicated a considerable O─H‐bond weakening of water upon coordination to the vanadium atom (BDFE(O─H)_avg_ of 62.4 kcal mol^−1^), suggesting that the coordination of water to reactive defect sites of metal‐oxide surfaces may enable water to act as a source of hydrogen atom. Kinetic isotope effect studies and Eyring analysis were consistent with the formal hydrogen‐atom‐transfer reaction from the aqua ligands to 1,4‐naphthoquinone, yielding complex **209** likely through a PCET pathway.

### 5.3 Tantalum

**FIGURE 43:**
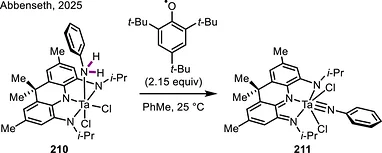
Aniline N─H‐bond weakening by tantalum complexes containing redox‐active ligands.

Abbenseth and coworkers developed a system based on tantalum(V) surrounded by an acridane‐derived redox‐active NNN pincer ligand, which enabled coordination‐induced N─H‐bond weakening of aniline (Figure 43) [129]. Both hydrogen atoms of the aniline NH_2_ group of **210** were cleaved with 2,4,6‐tris(*tert*‐butyl)phenoxyl radical, providing the corresponding imido tantalum(V) complex **211** with a two‐electron oxidized ligand. A DFT analysis provided BDFE values of **210** and the transient radical amido complex of 70 and 60 kcal mol^−1^, respectively, consistent with the observed two‐fold hydrogen‐atom abstraction.

### 5.4 Zirconium

**FIGURE 44:**
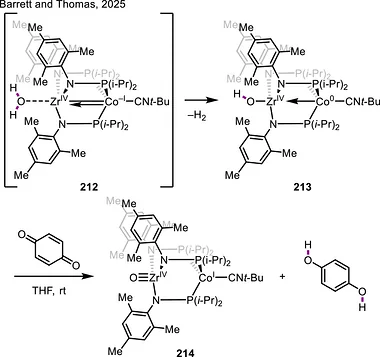
O─H‐bond weakening of water by bimetallic Zr─Co complexes.

Barrett, Thomas, and coworkers reported a heterobimetallic system consisting of redox‐inactive Lewis‐acidic zirconium(IV) and redox‐active cobalt(−I) metal centers that enabled O─H‐bond weakening of water (Figure 44) [130]. Coordination of water occurred only at the redox‐inactive Zr site, because of a tightly coordinated *tert*‐butyl isocyanate ligand sterically blocking the Co center. Spontaneous H_2_ evolution was observed upon addition of water from complex **212**. BDFE value of the O─H bond of the coordinated water was determined computationally as 43 kcal mol^−1^. The resulting [Zr]─OH complex **213** underwent a second hydrogen‐atom transfer to 2,4,6‐tri‐*tert*‐butylphenoxyl radical or *p*‐benzoquinone, providing zirconium(IV) oxo complex **214**. Open‐circuit‐potential measurements of **213** established the O─H‐bond BDFE value of 64 kcal mol^−1^, consistent with the value determined by DFT analysis and through reactions with stoichiometric hydrogen‐atom‐transfer reagents.

In a follow‐up study, Barrett and Thomas extended the application of the developed Zr/Co complex to N─H‐bond weakening of aniline [131]. 2,4,6‐Tris‐*tert*‐butylphenoxyl radical was used to abstract one or both hydrogen atoms of the coordinated aniline ligand, with experimentally determined BDFE values of 37 and 55 kcal mol^−1^, respectively.

## 6 Summary and Outlook

**FIGURE 45:**
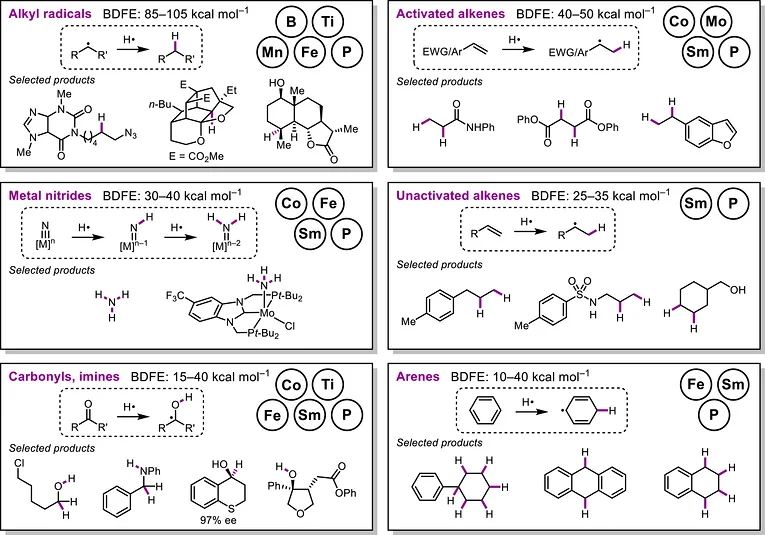
Overview of synthetic applications of protic molecules as formal hydrogen‐atom donors in reduction reactions promoted by redox‐active Lewis acids based on various central atoms.

This review has aimed to provide an overview of redox‐active Lewis acids that weaken the O─H, N─H, or S─H bonds of small protic molecules through in situ association. We focused on highlighting the diverse applications of these activated systems as formal hydrogen‐atom donors in organic synthesis (Figure 45). In addition, we sought to illustrate the broad range of both protic molecules and redox‐active Lewis acids capable of enabling such reactivity. We anticipate that many additional systems operating through this principle remain undiscovered, both in synthetic chemistry and in nature. Careful mechanistic investigations may help uncover such examples while also providing deeper insights into the physical origins of the bond‐weakening effect. Furthermore, a more detailed understanding of the mechanisms underlying the formal hydrogen‐atom transfer from these activated systems could guide the design of new platforms that suppress undesired side reactions, such as H_2_ evolution, while enabling selective substrate reductions. Challenging reductions of substrates such as N_2_ have been achieved using small protic molecules as a formal source of hydrogen atoms [66, 91, 92, 100, 123]. However, N_2_ activation was required for achieving the reductions. Development of more powerful but stable PCET reagents capable of direct reductions of N_2_ or CO_2_ represents an exciting area of future research.

The thermodynamic driving force for O─H and N─H‐bond weakening is typically associated with formation of a strong bond to O and N, respectively, in the by‐products formed from the redox‐active Lewis acid (e.g., P═O bond in phosphine oxides or Sm^III^─O bond in samarium oxides). Rendering these powerful systems catalytic will increase their efficiency and economy. Moreover, catalytic systems might also offer an advantage over stoichiometric reagents in mitigating competing H_2_ evolution by keeping a low concentration of the reactive formal hydrogen‐atom donor. The application of O─H/N─H‐bond weakening in enantioselective synthesis has been limited so far. The use of chiral redox‐active Lewis acids and/or chiral protic molecules could expand the repertoire of chiral hydrogen‐atom donors for enantioselective hydrogen‐atom‐transfer reactions, which could be especially useful in systems incompatible with thiol HAT catalysts. Future research in this area also holds potential for the discovery of new reactions that include a formal transfer of hydrogen atoms.

## Author Contributions

**Petra Vojáčková**: writing – original draft, writing – review and editing. **Armido Studer**: writing – review and editing.

## Conflicts of Interest

The authors declare no conflicts of interest.

## Data Availability

The data that support the findings of this study are available from the corresponding author upon reasonable request.
